# Granular evaluation of public primary healthcare accessibility in rural India

**DOI:** 10.1108/JHOM-02-2025-0065

**Published:** 2026-04-09

**Authors:** Archana Dang, Vastav Ratra, Damini Singh, Indrani Gupta

**Affiliations:** Institute of Economic Growth, Delhi, India; The London School of Economics and Political Science, London, UK; Indian Institute of Management Visakhapatnam, Visakhapatnam, India

**Keywords:** Healthcare accessibility, Spatial accessibility, Rural India, Primary healthcare, Public health infrastructure

## Abstract

**Purpose:**

India lacks a comprehensive, village-level assessment of primary healthcare accessibility needed to guide policies for improving access. This article provides a nationwide, village-level baseline measure of public primary healthcare accessibility in India using three distinct spatial metrics.

**Design/methodology/approach:**

A geocoded census of public healthcare facilities from the National Health Resource Repository is merged with spatial and demographic data for rural census villages. A multi-dimensional framework is developed to assess healthcare accessibility using three metrics: (1) a regional availability metric that captures infrastructure shortfalls relative to Indian Public Health Standards (IPHS) norms; (2) a measure using Euclidean distance to the nearest facility and (3) a capacity-constrained, catchment-based propensity-of-access metric conceptually aligned with the two-step floating catchment area method. Descriptive and spatial analyses are conducted at national and sub-national levels to highlight geographic variation in accessibility.

**Findings:**

The first metric shows that a rural Primary Health Centre (PHC) serves an average of 33,800 people, exceeding the Indian Public Health Standards norm of 30,000. The second indicates an average village-to-PHC distance of 5.49 kilometres. The third shows that, when population pressure and distance are considered jointly, residents in 20% of villages are effectively crowded out, even at the national average distance.

**Originality/value:**

This nationwide, village-level assessment is the first to integrate availability, proximity and capacity-adjusted access across India. The analysis challenges single-metric planning approaches and suggests that upgrading or expanding infrastructure alone cannot resolve persistent spatial and capacity gaps in rural healthcare. The insights extend beyond India, where similar metrics often misstate healthcare accessibility.

## 1. Background

Achieving Universal Health Coverage (UHC) entails the provision of affordable health services within reasonable distances to the population. In resource-constrained low- and middle-income countries, the location of government health facilities – the key providers of inexpensive healthcare – is a crucial determinant of population health. Distant, overcrowded, or low-quality public facilities may compel users to opt for private or alternative healthcare, which has implications for their health, out-of-pocket costs, and their propensity to utilize public health services in the future.

India, the most populous country in the world, has made some progress in improving healthcare accessibility over the past decades. The share of the population utilizing government-owned primary and tertiary healthcare facilities has somewhat risen between 2004 and 2014, but the private sector remains the provider of choice for most users (Jana and Basu, 2017). These trends have remained stable through the latest surveys in 2018 (National Sample Survey, 2019). The public sector has expanded its footprint in institutional deliveries, with its share rising from 21% of births in 2004 to 53% in 2014 (Joe *et al.*, 2018). Some of these gains are attributable to policy choices – schemes like Janni Suraksha Yojana (JSY) for institutional deliveries and insurance policies like Rashtriya Swasthya Bima Yojana (RSBY) have been key contributors to improvements. However, the supply of quality healthcare remains limited, with only modest gains in coverage of the public health footprint in the country. Between 2005 and 2022, for example, only 1700 additional Primary Health Centres (PHCs) were constructed (Rural Health Statistics – RHS, 2022), which would potentially cover only 33% of the increase in the rural population in this period. These problems are compounded by high vacancies, inconvenient timings and the unavailability of medical staff at primary care facilities (Chaudhury *et al.*, 2006), echoing similar challenges in primary care models in other developing economies like Ghana (Addi *et al.*, 2021) Furthermore, these supply constraints, combined with fragmented health financing systems – as demonstrated by constraints in Pakistan (Khan *et al.*, 2025) – exacerbate inequitable access by failing to protect remote and low-income populations from out-of-pocket costs. These financial and operational failures underline the structural fragility of public health systems in South Asia, including India.

Post-2018, the last year with reliable population-level health surveys in India, the government introduced a new scheme to revamp public healthcare facilities in the country. Notably, the government decided to revamp Health Sub-Centres (HSCs) and PHCs to create Health and Wellness Centres (HWCs). This involved upgrading the infrastructure of the PHCs and providing the staff with training, equipment and medicines necessary to address the Non-Communicable Diseases (NCDs), in addition to communicable diseases and maternal care mandates of primary facilities (National Health Systems Resource Centre – NHSRC, 2022). While these would make the facilities more useful for individuals suffering from a host of chronic conditions like hypertension and diabetes, it is unclear whether it would address problems of overcrowding at these facilities, especially since the initiative does not create many new HWCs. Instead, it supplements existing infrastructure with a greater workforce and resources, leaving physical accessibility relatively untouched. This is relevant because existing evidence suggests that, in maternal services alone, overcrowding at these facilities has dire consequences on selection into care, and maternal and infant mortality of the served population (Andrew and Vera-Hernández, 2024).

According to IPHS, the government should construct enough PHCs to ensure that a PHC serves no more than 30,000 individuals in non-hilly areas and 20,000 individuals in hilly areas. However, as of 2018, our analysis suggests that, on average, a PHC caters to 33,800 individuals. This has implications for people’s willingness to rely on public primary health services for their healthcare needs. In a survey of middle-aged and elderly individuals in India in 2017–2019, only 10% of those seeking outpatient care visited a PHC, a smaller fraction than those seeking outpatient care at pharmacies (13%) (Longitudinal Ageing Study in India – LASI, 2020).

Previous studies in India have largely relied on demand-side surveys, like the nationally representative survey conducted by the National Sample Survey Organization (NSSO), to understand the accessibility and availability challenges of public healthcare and its implications on health and finances of the populace (Mukherjee and Levesque, 2010; Jana and Basu, 2017; Srivastava and Gill, 2020; Mohanty *et al.*, 2022). The findings are unanimous – the share of public healthcare in the total market for primary care remains low, service provisions remain undependable and there is severe overcrowding with potential knock-on consequences. In some cases, focused surveys are used to assess demand-side factors of treatment seeking for specific diseases or geographies. For example, one study focuses on treatment-seeking behaviour among cancer patients in Odisha (Batra *et al.*, 2018), another study describes care-seeking journeys of individuals with chronic illnesses in select villages in India (Kane *et al.*, 2022). Additionally, another study examines oral healthcare-seeking behaviour among rural residents of a district in Maharashtra (Deolia *et al.*, 2020). These demand-side analyses have been crucial for documenting the consequences of supply-side issues, highlighting numerous self-reported barriers such as distance, cost and facility quality, particularly affecting women (Das *et al.*, 2023a, b). Furthermore, qualitative work has explored utilization challenges among hard-to-reach groups in specific areas like Kerala (Surendran *et al.*, 2024).

While these studies provide engaging insights into healthcare-seeking behaviour and its correlates, they are mostly unable to square demand-side responses with supply-side initiatives and policies launched by the government. Specifically, we have a poor understanding of the accessibility of healthcare infrastructure at the national level, and adherence of accessibility to norms laid down by the government and prescriptions provided by the international organizations.

Most papers on healthcare supply restrict their geographical focus to small areas, largely due to constraints on enumerating healthcare access. This is a large literature, and we do not attempt to enumerate it here. Broadly, however, these are primarily “case-study” style papers applying spatial metrics to limited units: for instance, recent geospatial analyses have been confined to the Midnapore municipality (in West Bengal), where they demonstrated that geographic barriers significantly exacerbate the consequences of health shocks (Das *et al.*, 2023), or focused on inequities within the state of West Bengal (Hasan and Ghosal, 2025). Similarly, researchers have looked at chronic care in a single district (Lall *et al.*, 2019), treatment in a single South Indian city (Ergler *et al.*, 2011), or a high-priority border district (Verma and Dash, 2020). Furthermore, while some work uses district-level spending data for granular equity analysis (Chatterjee and Smith, 2021), this data is available for only one of thirty states. Similar data is unavailable for other states in the country. Besides, some studies utilizing linked datasets like the Indian Human Development Survey (IHDS) and District-Level Health Survey (DLHS) provide a brief picture of randomly selected, nationally representative villages and healthcare facilities (Andrew and Vera-Hernández, 2024; Kulkarni *et al.*, 2023; George *et al.*, 2023). Such a restricted or incomplete geographical focus of the studies provides a limited understanding of the supply of healthcare for the entire country. Given the use of random sampling in these studies, we are only able to assess the spatial accessibility for less than 1% of all villages, which may not be useful for policymakers planning the expansion of primary healthcare facilities at the village level.

Internationally, GIS-based accessibility methods are increasingly used to evaluate spatial health inequities. Studies applying floating catchment methods – including recent public–private comparisons (Pérez-Fernández and Michel, 2025) – highlight the importance of integrating distance decay, facility distribution, and capacity constraints. Additionally, digital innovations such as telemedicine (Mwogosi, 2025) and artificial intelligence–enabled primary care tools (Lamem *et al.*, 2025) are proposed to address barriers in rural health systems. While these innovations may strengthen aspatial dimensions of access, such as quality and timeliness, they cannot substitute for a well-distributed physical network of primary care facilities. A spatial baseline remains essential for guiding the effective deployment of such technologies.

Collectively, the existing literature reveals a clear gap: prior studies examine either availability (facility counts), proximity (distance or travel time) or perceived access barriers, but none provide a unified, capacity-adjusted, village-level assessment of primary healthcare accessibility across India. No previous work uses a complete geocoded census of public health facilities, nor integrates population pressure, spatial proximity and IPHS-based capacity constraints. Our study addresses this gap using the National Health Resource Repository (NHRR) – the first full census of India’s public health facilities with geocoordinates – to conduct a comprehensive, pan-India, village-level analysis of rural primary healthcare accessibility, our first contribution to the literature.

Our second contribution to the literature is providing the first national-level application of this dataset to assess spatial healthcare accessibility and availability in rural India – an analysis not previously undertaken. The article incorporates three complementary measures – area-based, distance-based and catchment-based – drawing on regional availability, Euclidean distance and a measure conceptually related to the Two-Step Floating Catchment Area (2SFCA) approach.

Our empirical approach is grounded in an extensive theoretical literature that conceptualizes healthcare accessibility as comprising both spatial and aspatial dimensions. While aspatial factors, such as affordability, facility opening hours and cultural norms, play a critical role, our study focuses on spatial dimensions of accessibility. We first draw on Gravity and Spatial Interaction Models (Piovani *et al.*, 2018), which posit that service utilization rises with facility capacity and falls with distance. In addition, the Central Place Theory (Vionis and Papantoniou, 2019) informs our evaluation of spatial equity, whereby we measure straight-line distances from villages to the nearest PHC and compare these to idealized catchment patterns implied by IPHS norms. This allows us to identify micro-level pockets of under- or over-service that may be masked by district-level averages. Guided by the Equity of Access Framework (Levesque *et al.*, 2013), we compute for each village three interrelated metrics: *Regional Availability* (population-to-PHC or HSC ratio relative to IPHS standards), *Distance* (Euclidean distance to the nearest facility) and *Propensity of Access*, collectively yielding a policy-relevant spatial access profile. Finally, adapting the 2SFCA method (Wan *et al.*, 2012), we model the allocation of population demand to providers within a 14 km travel-informed catchment, again applying IPHS norms as binding capacity constraints. By aggregating each village’s weighted access to available PHC slots across its catchment, we derive a nationally consistent, capacity-aware metric that synthesizes supply, demand and spatial proximity into a unified measure of healthcare accessibility.

Below, we briefly describe each of these measures.

First, we assess *regional availability* – an area-based measure (Luo and Wang, 2003; Beks *et al.*, 2023) – by examining how the distribution of public health facilities aligns with population coverage norms under the IPHS. We perform this analysis at multiple geographic scales (national, state, district and sub-district) to highlight a key weakness of the current IPHS prescriptions – while the norms stipulate the number of health facilities for a certain population size, they do not define the spatial scale at which these ratios should be met. Thus, there is no clarity on whether the population-to-healthcare service ratios or norms should be met at the national, state, district or lower levels.

Second, we employ a *distance-based* approach (Beks *et al.*, 2023) by calculating Euclidean distances between population clusters and public health facilities. This enables us to move beyond prior datasets – such as the Census, Mission Antyodaya, and the Multiple Indicator Survey as they measure distance of a village from healthcare facilities in bins of 5 kilometres. We find that the average distances to key government health facilities are often lower or just around 5 kilometres. Thus, the precise way of measuring these distances in prior surveys masks key heterogeneities across villages. These heterogeneities are not trivial; recent research suggests that the marginal effect of additional kilometres is roughly linear even for short distances (McGuire *et al.*, 2021).

Third, we incorporate a catchment-based approach by accounting for population density and its interaction with facility proximity. This aligns with enhanced 2SFCA and gravity-based models that integrate both distance decay and supply-side constraints (Luo and Wang, 2003; Ma *et al.*, 2018; Beks *et al.*, 2023). Moreover, recent studies have applied geospatial modelling to assess accessibility using GIS-based methods (Das *et al.*, 2023; Pérez-Fernández and Michel, 2025), highlighting the importance of distance, facility distribution and population pressure. Our analysis shows that physical proximity alone does not ensure access – particularly in high-density settings where a small number of nearby users may absorb a disproportionate share of facility resources, effectively crowding out others within the same village/catchment. This demand saturation challenges the adequacy of one-dimensional planning norms and highlights the need for multi-criteria approaches that jointly consider spatial proximity, population pressure, provider capacity etc.

A recent national study by Patil *et al.* (2025) makes an important contribution by using friction-surface modelling to estimate travel times to PHCs and Community Health Centres (CHCs). While valuable, their approach focuses primarily on travel time and does not incorporate capacity constraints, IPHS norms or full national coverage. Our study complements this work by leveraging the NHRR – the first comprehensive, geocoded census of public health facilities – to develop a multi-dimensional assessment of rural healthcare accessibility. By combining regional availability, Euclidean proximity and a capacity-adjusted Propensity-of-Access index (based on a modified 2SFCA method), we provide the first granular, village-level, pan-India profile that captures both spatial deficits and population-driven crowding.

## 2. Material and methods

### 2.1 Data source

We combine three sources of data to conduct our analysis. For health facilities in India, we use the NHRR, which is India’s first-ever national healthcare facility registry with standardized and updated geospatial data of *all public and private healthcare facilities in India* (except West Bengal) for the year 2018. Central Bureau of Health Intelligence (CBHI) in consultation with the Directorate General of Health Services (DGHS), conceptualized the framework of making a health resources repository, where both public and private sector data were collected. Data were scraped from the NHRR website. The dataset covered both public and private facilities and included information on the type of facility (primary, secondary, tertiary) as well as the name of the village or town in which the facility is located. Further, for the population-related data at district, sub-district, town and village levels were obtained from the latest 2011 Census data using census website. We match the population census data with the NHRR data using village, sub-district, district and state names. The matching was carried out in Stata, and villages in NHRR that matched with multiple villages in the census were assigned to the match with the largest population.

We would like to mention that in India, official infrastructure statistics are released with a significant time lag, and no geocoded facility-level database comparable to NHRR 2018 is publicly available. RHS (2022–2023), now published under the new title Health Dynamics of India (Infrastructure and Human Resources) (2022–2023), shows only modest increases in HSCs and PHCs since 2018, indicating that rural infrastructure has been largely stable. The NHRR 2018 thus remains the only nationwide, facility-level dataset suitable for village-level spatial accessibility analysis.

To delineate hilly areas in India, we use the satellite on elevation, which is extracted from the Shuttle Radar Topography Mission (SRTM). These data are available at Socioeconomic High-resolution Rural-Urban Geographic (SHRUG) open data platform (Asher *et al.*, 2019).

### 2.2 Variables and measures

We elaborate upon three measures of healthcare accessibility used in this article – shortfall, distance and propensity of access.

#### 2.2.1 Infrastructure shortfall

As per the norms set by the government, a PHC is expected to serve 30,000 population in plain areas and 20,000 in hilly areas, while an HSC should serve a maximum of 5,000 in plain and 3,000 people in hilly areas (IPHS-HWC-SHC, 2022; IPHS-HWC-PWC, 2022). To assess whether these standards are being met, we examine the *regional availability* of public health infrastructure, using an area-based measure. We first calculate the total population served by a health facility at three different administrative divisions of India, namely, the subdistrict level, district level and state level. Specifically, if the population per HSC/PHC is greater than the prescribed population norm, then, it does not meet the norm. This approach reflects a classic area-based methodology, where adequacy is determined by comparing population-to-provider ratios within administratively bounded geographic units.

For HSC/PHC, we calculated the total population served by an HSC (PHC) by first dividing the total rural population by the total number of HSC (PHC) in a subdistrict/district/state. We then created a binary variable to indicate whether there is a shortfall in meeting the population norm by classifying subdistricts/districts/states into two groups, that is, one in which the total population served by an HSC (PHC) is below or at the population norm of 5,000 (30,000) given for HSC (PHC) in the plain areas and the other in which it is above this norm.

Since the population norm for HSC (PHC) is 3,000 (20,000) for hilly areas, we calculate the shortfall by dividing the administrative divisions into hilly and non-hilly areas. The Urban and Regional Development Plans Formulation and Implementation (URDPFI) Guidelines, given by the Ministry of Urban Development (Government of India), define hilly areas as any area above 600 m in height from mean sea level (URDPFI, 2015). We utilize the satellite data on elevation to delineate the hilly areas and calculate whether the norm is met in these areas by following the same approach as we do for the plain areas.

#### 2.2.2 Distance to nearest healthcare facility

As a *distance-based* measure of spatial accessibility, we compute the Euclidean distance (in kilometres) between the village whose accessibility is being measured from the nearest village that houses the healthcare facility under examination. While Euclidean distance does not account for variations in terrain, transportation infrastructure or individual mobility constraints, it remains a widely used proxy for physical accessibility in large-scale spatial analyses. By abstracting from these regional heterogeneities, this measure enables a uniform, nationwide comparison of accessibility. It is often the case that larger Euclidean distances co-exist with poorer infrastructure access.

#### 2.2.3 Propensity of access to a primary healthcare facility

To capture spatial healthcare accessibility beyond simple proximity, we devise a propensity of access metric that simultaneously accounts for both distance and population-based service capacity. This catchment-based measure reflects the probability that a village might be served by a PHC within a reasonable geographic range, given population constraints.

To devise this metric, we create circular hinterlands of 14-km radius around the PHCs in our sample. We define the villages lying in this hinterland as the potential clients of a PHC. A village, hence, may lie within the hinterland of a single PHC, multiple PHCs or may lie outside the reach of any PHC. As a corollary, villages with the nearest PHC more than 14 kilometres away would not be allotted to any PHC, thus their propensity for access is zero.

The choice of hinterland is dictated by secondary data on the health-seeking behaviour of rural individuals. The source of relevant data is the Longitudinal Ageing Survey of India, conducted between 2017 and 2019. Limiting our sample to middle-aged and elderly individuals living in rural areas, we find that 90% of individuals visiting a PHC for their last outpatient visit lived over 14 kilometres from the PHC they visited. Thus, since a PHC mostly serves individuals living up to 14 kilometres away, we consider that as the hinterland of a PHC. We recognize that the radius of hinterland is an endogenous parameter – reducing distances to PHCs would also reduce the size of hinterland – but it provides a useful frame to illuminate the access that residents of distant villages may have to rationed public services like health establishments.

According to IPHS, every PHC must only serve a maximum of 30,000 individuals. Once we have created the hinterlands, we match PHCs and villages such that each village is allotted to a single PHC and each PHC serves no more than 30,000 individuals, starting with the individuals located in the villages closest to it. This matching exercise yields multiple permutations of village-PHC matches. To compute the propensity of access – the share of permutations in which a village is matched to any PHC – we repeat this exercise 50 times. In effect, our approach is a random sampling over all permissible PHC-village permutations.

We consider a village as allocated if it is allotted to a PHC in an allocation simulation. The proportion of simulations in which a village is assigned to a PHC forms the propensity of access of the village. We generate variation in access propensities by randomizing the order of matching. We do this to circumvent the problem of solving an optimization problem that assigns each village to a PHC, accounting for all other village assignments. The propensity of access provides, here, a measure of accessibility to nearby PHCs (not just the nearest PHC) when local population density and wider geographical primary healthcare footprint are considered.

Our Propensity of Access measure builds on the conceptual foundations of the 2SFCA method, which seeks to capture spatial accessibility by accounting for both geographic proximity and supply-side constraints. Like 2SFCA, our approach defines fixed-radius catchments (14 kilometres) around each public health facility and allows villages to fall within multiple facility hinterlands. Both methods recognize demand competition by considering the population size served within each catchment. While enhanced 2SFCA explicitly models distance decay (e.g. using weights), propensity of access implicitly incorporates distance by starting the allocation with the nearest villages — closer villages are more likely to be served. Rather than calculating a deterministic score, we simulate multiple capacity-constrained allocations of villages to nearby PHCs to estimate the probability that a village is served. While not a substitute for more formal optimization models, this approach offers a tractable and interpretable tool for assessing access under capacity constraints in rural health systems.

## 3. Results

### 3.1 Sample characteristics

Currently, the NHRR database contains the location of over 10,18,891 health establishments. The scraped data obtained from the NHRR website in late 2022 had data on 901,899 health establishments and covers more than 85% of HSC and PHCs. We combined this data with the geocodes of villages from the World Bank and cities from Google Maps to create a geo-referenced dataset for later calculations.

The NHRR scraped data were at the health establishment level and they were brought to the village/ward level, which resulted in 190,019 village/ward observations. The 2011 census data at the village/ward level were utilized and had 665,074 observations. Out of 665,074, 586,497 were villages and 78,577 were wards. Out of 586,497, 142,000 villages matched with the NHRR data. We assumed that the remaining villages do not have any health establishment.

### 3.2 Regional availability measured based on norms

Using an area-based measure of regional availability, we assess the extent to which the distribution of public health infrastructure aligns with population-based coverage norms prescribed under the IPHS. This approach evaluates whether healthcare facilities are sufficiently available across administrative units, relative to the rural population they are intended to serve.

According to our analysis, the national-level estimation reveals that one PHC provides care to 33,800 people in rural areas. A regional disaggregation reveals deeper gaps in regional availability: at the subnational level, we find that 44% of states and roughly 59% of districts and sub-districts do not adhere to the government’s standards (Table 1).

**Table 1:** Proportion of areas not meeting the norms for Health Sub-Centres (HSC) and Primary Health Centres (PHC) in rural areas at different levels

Administrative units	HSC	PHC
Sub-districts	68.6%	58%
Districts	71%	58.8%
States	73.5%	44%

**Note(s):** 5,000 and 30,000 population norms for HSC and PHC, respectively, are used for the plain areas, while 3,000 and 20,000 population norms are used for hilly areas

**Source(s):** Authors’ own work using NHRR, 2018 and population census, 2011

A similar shortfall is observed for HSCs. At the national level, one HSC serves 6,086 rural population. In addition, we find that 71% of districts and 68% of sub-districts, respectively, are underserved by HSCs, and that 74% of states do not meet the necessary standards for HSCs (Table 1).

**Figure A1:**
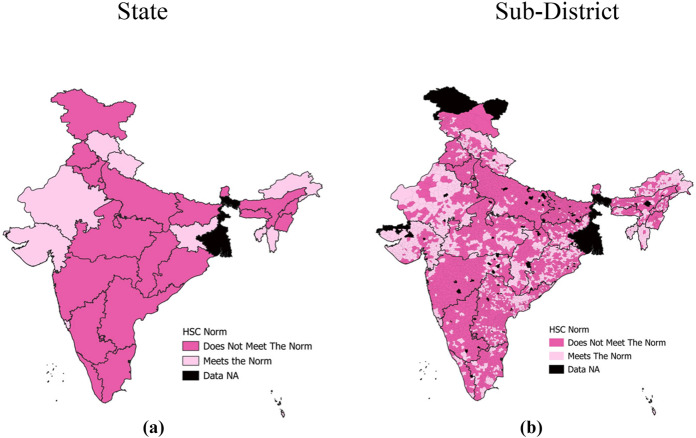
HSC norm, sub-national variation. *Notes*: These maps show the distribution of areas that meet the government-prescribed norm for HSC at (a) the state level and (b) the sub-district level. Areas in black represent states whose data are missing. Source: Authors’ calculations

To further unpack these disparities, we disaggregate the analysis by Empowered Action Group (EAG) and non-EAG states (Table 2). EAG states are a group of eight less developed states of India, which account for the majority of the under-five mortality burden of the country (Mani *et al.*, 2012; Arokiasamy and Gautam, 2008). These are: Rajasthan, Uttar Pradesh, Uttarakhand, Bihar, Jharkhand, Madhya Pradesh, Chhattisgarh and Orissa. Greater disparities in the availability of HSC and PHC across EAG and non-EAG states are shown in Table 2, which demonstrates that the proportion of sub-districts not fulfilling the requirements for both HSC (72%) and PHC (71%) is significantly higher for these states, especially for PHCs. As these states correspond with areas with lower access to all-weather roads and transportation infrastructure, the effects of infrastructure shortage might be compounded by the absence of complementary infrastructure (see Figure A1 in Appendix). While area-based population norms offer a starting point for planning, they may obscure deeper spatial inequalities – especially when not evaluated alongside distance, density and transport access.

**Table 2:** Proportion of sub-districts not meeting the norms for Health Sub-Centres (HSC) and Primary Health Centres (PHC) by Empowered Action Group (EAG) and non-EAG States

Type of facility	EAG	Non-EAG
HSC	71.9%	65.6%
PHC	71.4%	47.3%

**Note(s):** 5,000 and 30,000 population norms for HSC and PHC, respectively, are used for the plain areas, while 3,000 and 20,000 population norms are used for hilly areas. EAG states include Rajasthan, Uttar Pradesh, Uttarakhand, Bihar, Jharkhand, Madhya Pradesh, Chhattisgarh and Orissa

**Source(s):** Authors’ own work using NHRR, 2018 and population census, 2011

### 3.3 Accessibility measured by distance

Using the coordinates of all villages in India, we estimate distance-based measures of spatial accessibility by calculating the Euclidean distance from each village to the nearest village with a public healthcare facility, as described in Section 2.2.2. We report the first cross-country estimates of healthcare accessibility in Table 3. It should be noted that the nearest healthcare facility may not be the modal healthcare facility used by the village, and there may be some concerns about the operationality of the facility.

**Table 3:** Average Distance (in kilometres) and proportion of villages in different distance categories by Public Healthcare Facilities in India

Type of healthcare facility	Mean/Share	Standard deviation
*Health Sub-Centres (HSC*)	2.4 km	3.3
*D* ≤ 1 km	24.3%	
1 km < *D* ≤ 5 km	67.1%	
5 km < *D* ≤ 10 km	7.0%	
*D* > 10 km	1.7%	
*Primary Health Centres (PHC)*	5.5 km	4.8
*D* ≤ 1 km	5.0%	
1 km < *D* ≤ 5 km	50.9%	
5 km < *D* ≤ 10 km	33.7%	
*D* > 10 km	10.6%	
Community Health Centres (CHC)	11.3 km	8.5
District Hospital (DH)	17.3 km	14.2
Government Tertiary Hospital (GTH)	8.6 km	6.2

**Note(s):** The results are reported for *N* = 584,674 villages located in 35 states and Union Territories of India (excluding West Bengal). The distances are Euclidean distances that only consider the distance between the centroids of villages, and do not account for intra-village population distribution

**Source(s):** Authors’ own work using NHRR, 2018

We make three key observations. First, the median distance to the HSCs – the first line of primary care referral in India – is around 2.4 kilometres, corresponding to a walking time of around half an hour. However, while the HSCs were supposed to be the first point of contact and referral, they are seldom used for healthcare in India, with less than 3% of the middle-aged and elderly rural population reporting a visit to an HSC in 2017–2019 (authors’ calculation from LASI 2017–2019, rural sample). Second, the PHCs – the more widely used primary care facility – are 5.5 kilometres away from a village, on average. Roughly 1 in 10 villages have a PHC within their village. Third, the secondary and tertiary care facilities are furthest away, between 8 and 17 kilometres from the villages in our sample. This classification, and the corresponding findings, align closely with results on healthcare accessibility from Sub-Saharan Africa (Falchetta *et al.*, 2020).

**Figure 1:**
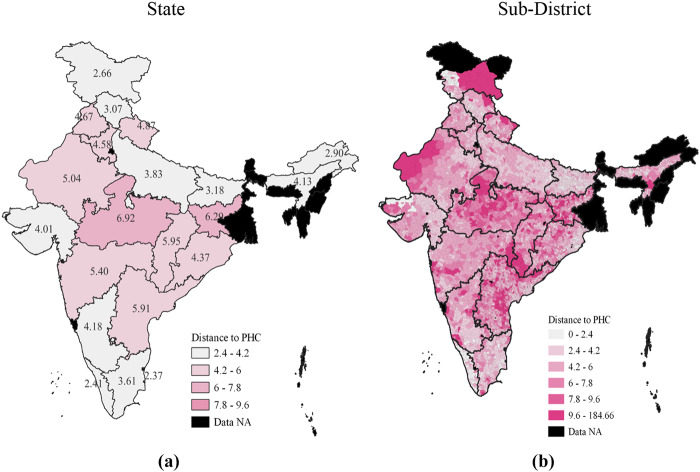
Distance to Primary Health Centres (PHC) by state and sub-district: *Notes:* Distances are computed from the centroids of villages to the centroid of the village/town with the nearest PHC. We restrict ourselves to the median instead of the mean value for these maps. The map in figure (a) reports the median distance to PHC from a village for a state, while the map in figure (b) reports the same for a sub-district. Areas in black represent states whose data are either missing in the original source (West Bengal) or are excluded from analysis due to their small size (the rest). We have synchronized the scales across maps; thus, the darkness of colour represents the same variation in distance in the two maps. *Source:* Authors’ own work using NHRR, 2018 and population census, 2011

In succeeding sections, we restrict our analysis to the PHCs because of their importance in healthcare provision. The averages in Table 3 may mask large disparities across geographies in India. To unpack these, we show the state-wise and sub-district-wise distance to the nearest PHC in Figure 1. There are two levels at which these inequalities exist. First, there are stark differences between states, where the average distance to PHC ranges from around 2 kilometres in Himachal Pradesh to nearly 7 kilometres in Madhya Pradesh. These differences are a direct result of policy differences as states, according to the Constitution, shoulder the responsibility of providing primary healthcare to citizens. Second, there are substantial differences within states. In Rajasthan, for example, there is a clear gradient in availability as one reaches sub-districts closer to the international border. Similarly, in Chhattisgarh, the areas with large forest cover in the southern parts of the state have larger average distances than their counterparts in the North. These are potentially driven by political-economic factors that govern the allocation of limited resources across villages in the state.

This distance-based measure complements area-based availability assessments by offering a geographically grounded view of physical access and emphasizing the role of spatial inequalities in shaping health service delivery across India.

### 3.4 Accessibility measured by propensity of access

While distance-based measures proxy geographic accessibility and regional availability norms capture infrastructural shortfalls, each reflects only one dimension of healthcare access. To provide a more comprehensive measure, we compute the propensity of access – a probabilistic, catchment-based metric that accounts for both spatial proximity and population-based capacity constraints, as detailed in Section 2.2.3. This cumulative measure reflects the likelihood that a village is effectively served by a nearby PHC, given distance, service capacity and competing population demand.

**Figure 2:**
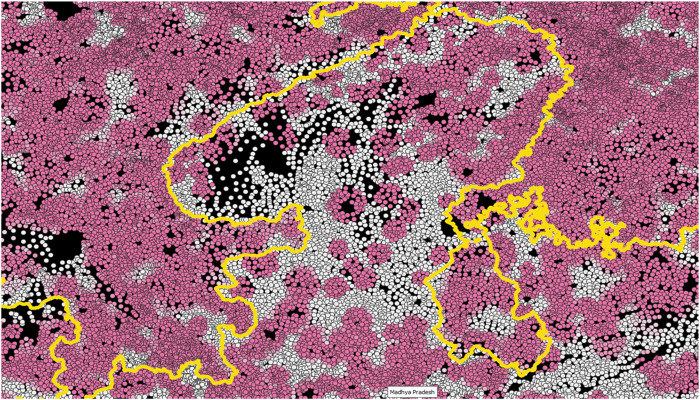
Propensity of Access, Sample Map: *Notes:* This figure shows the distribution of propensity of access across villages – represented by dots – in Northern Madhya Pradesh, India. Scale has been excluded for brevity and redundancy. Darker shades of pink correspond to villages with greater access to a Primary Health Centre (PHC), while the yellow boundary represents the state borders. *Source:* Authors’ own work using NHRR, 2018 and population census, 2011

Figure 2 is an illustration of allocation shares from northern Madhya Pradesh and some adjoining areas, and shows the consequence of this allocation. This area is representative of the patterns of propensity of access across the states in the sample. The darkness of the dots – each representing a village – represents the propensity of access.

**Figure 3:**
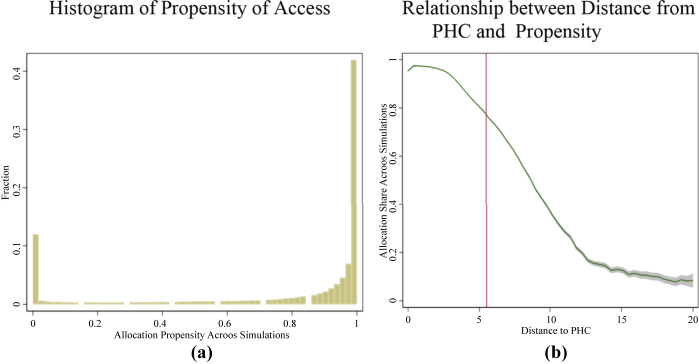
Propensity of Access, Distribution: *Notes:* Figure (a) plots the distribution of this measure across villages, and notes that 40% of villages are firmly allocated to a PHC across simulations and another 11% are never allocated. The remaining are occasionally unallocated, but there is a stark divide between the two, signalling a clear existence of underserved areas. Figure (b) plots a local polynomial curve that shows how the average propensity of access varies by distance from the PHC. *Source:* Authors’ calculations using NHRR, 2018 and population census, 2011

Two points can be noted from this figure. First, as is also shown in Figure 3, the simulation makes a clear distinction between allocated and unallocated villages. Large swathes of areas are systematically underserved, as opposed to a version where every area has overcrowded health facilities. If the latter were true, then all villages would have had a uniform shade of light pink, indicating low distances but a highly variable crowd at the health facilities.

In practice, however, the large underserved areas, visible as swathes of whiteness in Figure 2, are served by whichever facility seems nearest to the residents. This translates into the crowded status quo that characterizes public primary healthcare in India. We discuss two other caveats to this description in Section 4.

Second, despite sensible average distances, accounting for population substantially reduces the propensity of being adequately served by a PHC. A few villages located near the PHC may exhaust their resources, leaving little room for the treatment of residents of distant villages. To show this, Figure 3 plots the average propensity of access against distance from the village. Even at a distance of 5.5 kilometres – the average distance from a PHC – the propensity of access calculations suggests that more than 20% of the villages would remain unserved. The decline is exceptionally steep, and the propensity falls below 50% by 8 kilometres. This suggests that, accounting for density, small distances offer no guarantee of healthcare accessibility.

Catchment-based measures offer a fuller picture of functional access, factoring in distance, capacity, and population demand. The propensity of access goes beyond proximity to capture the real likelihood of service use – offering critical insights for equitable health planning and equitable healthcare delivery, as discussed in Section 4.

## 4. Discussion

India’s public primary healthcare system – the backbone of its maternal and child health programs, pandemic preparedness and prevention systems, and the first point-of-contact with health services for millions of Indian poor – has witnessed significant expansion since the Bhore Committee Report and the First Five Year Plan of 1952. The Bhore Committee in India crafted the three-tier health care system in 1946 with the principle that services should be located as close to the people to ensure maximum utilization. The strategy was primarily designed with the rural population in mind, as the committee was aware of the dire state of public health in India’s rural areas (Duggal, 1991). Compared to rural areas, cities and developed states have always had greater geographic access to healthcare services (Kumar, 2004). While these facts are well-known at the national level, relatively little information exists regarding differences at the sub-national level.

Despite the centrality of primary care in India’s health system, no systematic assessment of public healthcare accessibility in rural areas has been undertaken to date. This study fills that gap by offering the first comprehensive analysis of spatial accessibility and availability of public primary healthcare in rural India. Leveraging scraped data from the NHRR – a complete facility census – and population data from the 2011 Census, we evaluate three dimensions of access: (1) regional availability, measured against IPHS norms; (2) distance-based access, using Euclidean distance to the nearest facility and (3) catchment-based access, measured through the propensity of access accounting for competition and facility capacity. These metrics are computed across national, state, district and sub-district levels.

Based on IPHS for public facilities – our first metric – rural populations are underserved; that is, one PHC serves 33,800 people, above the recommended maximum of 30,000 individuals. Around 44% of states do not meet the IPHS requirements for PHC. The Rural Health Statistics (RHS) is another source that offers some accessibility indicators for India’s rural areas. According to the RHS 2018–2019, the national estimate is 35,557, which indicates that the rural population is underserved and that almost 32% of the states do not satisfy these requirements (RHS, 2019). Even though the estimates are comparatively similar, some limitations of our data source, listed later, may contribute to the mismatch in the estimates. Importantly though, RHS does not provide such estimates at the district and sub-district level, thereby underscoring the importance of our study.

The second measure – the distance between the village and the primary health centre – serves as our distance-based metric. We find that the average distance travelled by people to get to the closest PHC is 5.5 kilometres, an estimate that is close to the World Health Organization (WHO)’s recommended 5 km distance to the nearest health facility (Perks *et al.*, 2006). However, the average radial distance a PHC covers is 6.18 kilometres, according to the RHS 2019. In addition, the average distance differs between states; in Himachal Pradesh, for example, it is 2 kilometres, while in Madhya Pradesh, it is 7 kilometres. Similar variations exist within states; in certain Rajasthan sub-districts, for instance, residents must travel more than 10 kilometres to receive public primary care.

Our catchment-based metric – the propensity of access – reveals that over 20% of the villages located at an average distance of 5.5 kilometres from a PHC may not benefit from healthcare provision because they might be crowded out by individuals living closer to the facilities. This implies that shorter distances do not ensure healthcare accessibility, when population density is taken into consideration, leading to overcrowding and long wait times at healthcare facilities. The average propensity of access varies across states for instance – over 48% remain unserved in Jharkhand, while in Kerala this proportion is 8%.

Nonetheless, there are certain limitations of the study. As no census has been conducted since 2011, we had to use information from the 2011 census to determine accessibility using population-based norms. Moreover, population projection data are unavailable at both the sub-district and district levels. Our estimates, if any, are underestimates of the true parameters due to the increase in population since 2011.

Measuring healthcare accessibility using distance to the healthcare facility has at least three limitations. First, we do not know the exact location of the health facility, but we use the centroid of the village containing the health facility as a proxy for location. A more thorough dataset that accounts for the exact location might yield different results, but *ex ante*, the direction of the bias is not obvious. Importantly, it seems likely that the magnitude of correction, if any, would be an insignificant fraction of the distance to health facilities.

Second, we use a slightly different definition of PHC while computing results for norm-based and distance-based accessibility. While calculating the distance to the nearest PHC, we also include the Urban PHCs, Urban Health Posts, and Urban Family Welfare Centres in our sample, as these may be closer than the rural PHC for certain villages. As there are no restrictions on which PHC can be used by individuals, it would appear prudent to include relevant suppliers of primary care in the sample. This is unlike the norms-based calculations, where the government’s own policies mandate the provision of separate and sufficient infrastructure across rural and urban regions. To the extent that our results are affected by this decision, our distances reflect an over-optimistic measure of access. While these urban facilities represent a third of primary care facilities labelled as PHC in our analysis on distance, the overall effect on the result is limited by their rather proximate location within cities, thereby localizing the impact to a few villages in the immediate vicinity of cities.

Third, the Euclidean distances reported in the article may not appropriately account for geographical, climatic, infrastructural or social variegates in the sample. The effect of distance on accessibility is mediated through the kind of transportation and road infrastructure available between the village and the health facility, and whether this infrastructure is resilient to climatic and seasonal shocks. This again biases our results towards an optimistic approximation, underscoring that the scale of issues in healthcare access on ground is, if anything, worse than our estimates would show.

In addition to the limitations of distance and norm-based methods for measuring healthcare access, the propensity of access suffers from two other limitations. First, our measure of the radius of the hinterland is an endogenous parameter and is itself a function of healthcare accessibility. This is not necessarily a troubling concern, however, as most PHCs in our sample end up exhausting their quota of 30,000 individuals sufficiently before reaching 14 km. In practice, hence, the limit is only valid for a vanishing fraction of allocations.

Second, one must acknowledge the large literature on active patients in developing countries. The primary, secondary, and tertiary facilities offered by central, state, and local administrations are underutilized in rural areas, and many “active patients” bypass closely located and cheaper public health facilities to seek care at private hospitals located further away (Leonard, 2014). This may imply that assuming the same hinterland for all PHCs, irrespective of their staffing patterns and quality, might not align with treatment-seeking behaviour. It also does not account for the presence of private health facilities, and patient’s preferences over opting for them. These weaknesses, however, do not detract from the governmental policy and mandate to provide a functional PHC for every 30,000 rural residents, which it currently fails to do.

Finally, in any of these measures, we do not account for the quality of healthcare provision, disease burden or suitability of care. Our dataset, extensive as it might be, is incapable of addressing these pressing concerns about healthcare provision in India. A reasonable sequel to this study would involve surveying health facilities and monitoring the availability of healthcare professionals and medicines over time, as done by a recent study (Dreze *et al.*, 2024). Researchers in the future may use this data as a sampling frame to conduct sampling exercises necessary to advance the literature on these fronts.

### 4.1 Implications and conclusion

This is the first study to provide spatial analysis of public primary healthcare accessibility in rural India, using a newly geocoded census of health facilities linked with village-level demographic and geographic data. By combining population-based norms, distance-based proximity, and catchment-based metrics of functional access, we offer a comprehensive and multi-dimensional understanding of rural healthcare access. Our findings reveal stark shortages and spatial inequities, with PHCs often located far from villages and overwhelmed by demand – particularly in underserved regions.

The National Health Mission (NHM), the nation’s flagship program for building health systems, was established with the goal of improving access to equitable, affordable and quality health care, which is accountable and responsive to the needs of people. The National Health Policy of 2017 called for allocating two-thirds of the health budget to primary health care and suggested bolstering the delivery of primary care by creating HWCs as the foundation for providing comprehensive primary care. As the cornerstone of Ayushman Bharat, the central government stated in February 2018 that 150,000 HWCs would be developed by converting current Sub-centers and Primary Health Centres. The NHRR mapping conducted in 2018 indicates that there are roughly 152,000 HSCs and PHCs. Although the government declared that these will be converted to HWCs, the policy on constructing new primary care clinics or HWCs in underserved areas remains unaddressed. According to the government’s criteria, our analysis indicates that around 58% of districts and sub-districts are underserved. The first implication is that new PHCs and HSCs – or HWCs – are needed in the underserved parts of the country. Thus, merely upgrading existing facilities is insufficient – there is an urgent need to establish new facilities in areas with inadequate coverage to move closer to universal, equitable access.

Second, the government does not specify the administrative units to which population norms prescribed by the IPHS can be applied. That is, while we know that a PHC should serve 30,000 individuals, the implications of this norm for the number of requisite PHCs at the sub-national level remain unclear. At times, while the norm is adhered to at the state level, districts within the state might have lower access to PHCs than prescribed by the norm. We implemented the government-prescribed norms at every administrative level and found variation in estimates. Therefore, the second implication is that the government must specify the spatial scale (administrative unit) to which these standards are applicable. This is critical for informed planning and effective allocation of public resources, especially if the marginal gains to access vary across regions and population health needs.

Third, our findings underscore the limitations of relying solely on population-based availability norms. While such metrics are commonly used, they do not necessarily align with measures of geographical accessibility (e.g. distance) or functional access (e.g. propensity to be served, accounting for facility capacity and competition). Consistent with the literature (e.g. Lawal and Anyiam, 2019), our results emphasize the need to treat accessibility as a multi-dimensional concept – one that integrates spatial, infrastructural and service-delivery realities. The implication is clear: facility planning must jointly account for both population and distance, recognizing that proximity alone does not ensure access, especially in high-density settings where service capacity is easily exhausted. Moreover, recent studies have applied geospatial modelling to assess accessibility using spatial methods (Das *et al.*, 2023; Pérez-Fernández and Michel, 2025). While these efforts highlight the importance of distance and facility distribution, they also underscore the need for sophisticated approaches – like the 2SFCA methods – to capture the effects of population pressure and capacity constraints on access.

Our article contributes methodologically and empirically to the spatial health literature in India. Methodologically, it develops a three-part framework for measuring access to public primary healthcare that integrates IPHS norms, village–facility distances and a capacity-constrained, catchment-based propensity of access measure. Empirically, it uses the NHRR, which remains the only nationwide geocoded census of all the public facilities. This framework can be re-estimated whenever newer geocoded facility data becomes available, so it is not tied to a single year. It provides avenues for researchers for future research into the socio-economic determinants (aspatial) of healthcare inaccessibility and the supply-side drivers of health outcomes and further probes the supply-side determinants of population health in India. Policymakers will benefit from these findings and can potentially adopt a different approach to facility planning that accounts for both population and distance.

For policymakers and managers, the results provide a practical tool to identify underserved clusters and to prioritize where new or upgraded PHCs and HWCs are most needed, rather than relying only on norms or distances in isolation.

## Figures and Tables

**Figure A2:**
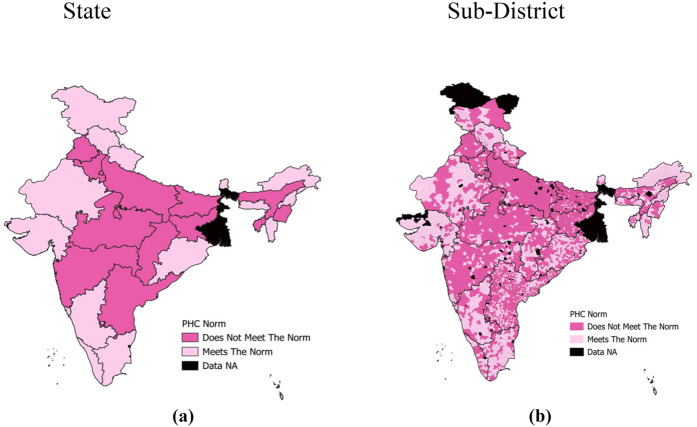
PHC norm, sub-national variation. *Notes*: These maps show the distribution of areas that meet the government-prescribed norm for PHC at (a) the state level and (b) the sub-district level. Areas in black represent states whose data are missing. Source: Authors’ calculations
